# First person – Ilaria Gregorio

**DOI:** 10.1242/dmm.049855

**Published:** 2022-09-21

## Abstract

First Person is a series of interviews with the first authors of a selection of papers published in Disease Models & Mechanisms, helping researchers promote themselves alongside their papers. Ilaria Gregorio is first author on ‘ Collagen VI deficiency causes behavioral abnormalities and cortical dopaminergic dysfunction’, published in DMM. Ilaria conducted the research described in this article while she was a PhD student in Paolo Bonaldo and Matilde Cescon's lab at the University of Padova, Italy. Ilaria is now a research associate in the lab of Botond Roska at the Institute of Molecular and Clinical Ophthalmology Basel, Switzerland, investigating vision and eye diseases, and developing new therapies for vision loss.


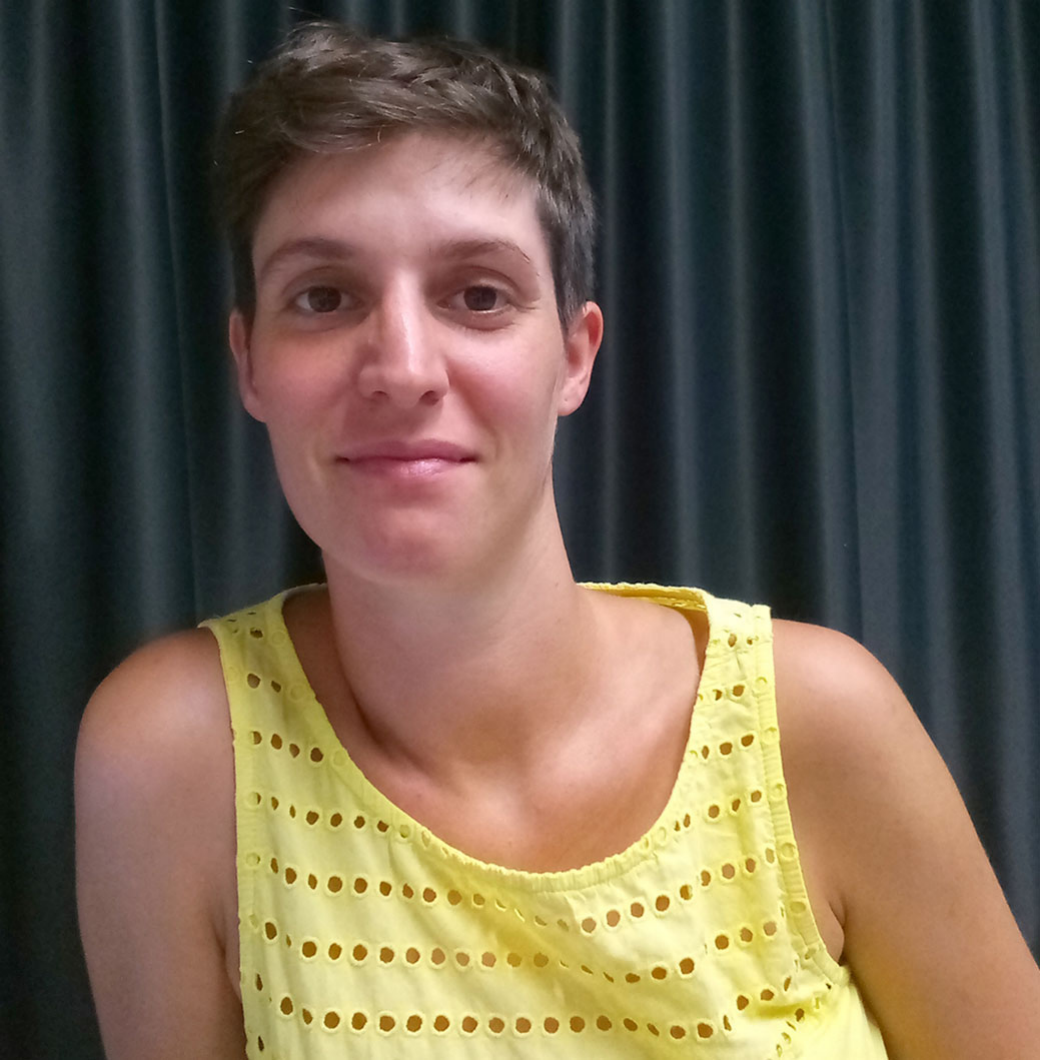


**Ilaria Gregorio**

**How would you explain the main findings of your paper to non-scientific family and friends?**

Bethlem myopathy (BM) and Ullrich congenital muscular dystrophy (UCMD) are genetic diseases caused by mutations in the protein collagen VI. As the names suggest, these diseases primarily affect the skeletal muscles, and related tissues such as tendons and nerves. Given that collagen VI was previously found to also have a role in non-muscular tissues, including the ageing central nervous system, we used mice lacking collagen VI, which mimic the mentioned human diseases, and analysed for the first time their behaviour at a younger age. Surprisingly, we observed altered levels of the neurotransmitter dopamine and anomalous behaviours in specific cognitive areas. Driven by these findings, we designed a special, ad hoc panel of neuropsychological tests to submit to a group of BM and UCMD patients, and again found low performances in cognitive areas linked to those affected in the mouse model. Finally, we found that collagen VI might have a role in the correct maturation of a subset of neurons – dopaminergic neurons – which could explain the anomalous behaviour of the mouse model and the distinct cognitive performances in the patients.

“Our study highlights for the first time the presence of a new set of signs linked to collagen VI myopathies that involve the cognitive domain.”

**What are the potential implications of these results for your field of research?**

Collagen VI-related myopathies are rare diseases with a broad range of severity. The most severe symptoms are certainly progressive muscle weakness and joint contractures that interfere with the ability to walk and can lead to life-threatening respiratory failure. However, our study highlights for the first time the presence of a new set of signs linked to collagen VI myopathies that involve the cognitive domain. We think it is important that the cognition and psychological features of patients with these diseases are evaluated and taken care of by physicians to improve their quality of life, already hampered by the joint contractures and muscular weakness.

**What are the main advantages and drawbacks of the experimental system you have used as it relates to the disease you are investigating?**

A great advantage of using mouse models in neuroscience is their similarity with humans: the genes involved in brain development and functioning are almost the same, and the molecular and cellular pathways operating in rodent and primate brains are highly conserved. However, although our collagen VI-knockout mouse model properly recapitulates many features of the disease, it obviously cannot perfectly mirror all the heterogeneous symptomatology of collagen VI-related myopathies. That is why it was so important for us to confirm our observations with a translational approach, involving a cohort of collagen VI patients.

**Figure:**
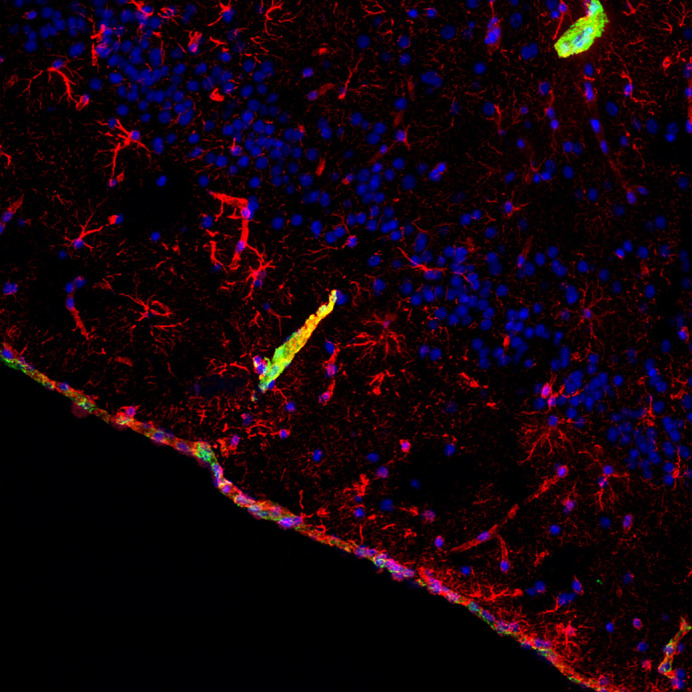
Astrocytes (red) and collagen VI (green) localized at the meninges and around blood vessels in the mouse brain.

**What has surprised you the most while conducting your research?**

What surprised me the most was to observe that collagen VI, a protein with little to no deposition in the brain parenchyma but only in the connective tissue associated with it (such as the meninges and blood vessels), can have an influence in such specific cognitive domains. This tells us one more time how complex and unexpected the functions of extracellular matrix components can be.

**What do you think is the most significant challenge impacting your research at this time and how will this be addressed over the next 10 years?**

I think that one of the challenges impacting collagen VI research is its complexity. It is an intricate and large protein, made of different chains that undergo several post-translational modifications. Its interaction partners are many, they differ depending on the tissue and probably lots of them are still obscure. All these features make collagen VI signalling extremely difficult to study, which is one of the reasons why no targeted therapies for collagen VI-related diseases have so far been developed. However, with further research and the advancement of new technologies, spanning from biochemistry to animal transgenesis, the biology of this protein will be further uncovered. This will hopefully bring more substantial help to patients.

**What changes do you think could improve the professional lives of scientists?**

In my opinion, young scientists are not well informed about non-academic career paths and about the potential of their transferable skills. This often leads to young researchers accepting fixed-term positions that eventually leave them frustrated and unmotivated. To avoid this, I think that undergraduate and PhD students should be mentored about the importance of jobs outside academia, including entrepreneurial professions, to help them find their right career path.

**What's next for you?**

After my PhD, I joined the Institute of Molecular and Clinical Ophthalmology in Basel as a research associate, where I work with other basic scientists and clinicians to understand vision and its diseases.
